# Manufacture and Characterization of Polypropylene (PP) and High-Density Polyethylene (HDPE) Blocks for Potential Use as Masonry Component in Civil Construction

**DOI:** 10.3390/polym14122463

**Published:** 2022-06-17

**Authors:** Taiza Ferreira, Gleisson Amaral Mendes, Andrielli Morais de Oliveira, Carmen Gilda Barroso Tavares Dias

**Affiliations:** 1 Institute of Technology, Federal University of Pará, Street Augusto Corrêa, 01, Belém 66.075-110, Brazil; gmendes@uepa.br (G.A.M.); cgbtd@ufpa.br (C.G.B.T.D.); 2 LABITECC, School of Civil and Environmental Engineering, Universidade Federal de Goiás (UFG), Goiânia 74.605-220, Brazil; andriellimorais@ufg.br

**Keywords:** plastics materials, sustainable construction, polyolefin blocks, civil construction, rotational molding

## Abstract

The lack of suitable destinations for plastics materials can be a global environmental problem. The alternative use of materials for sustainable construction encourages the standardization of waste and promotes effective social, environmental and economic gains at the local level and ensures savings and income for communities. The aim of this paper is the development, manufacture, and characterization of PP and HDPE recycled polyolefin blocks as masonry components in civil construction. These blocks were manufactured by the rotational molding process. Besides this, the mechanical, physical, impact and flammability properties of the blocks were studied. In conclusion, HDPE showed better behavior than PP in tests realized.

## 1. Introduction

Overall, plastic materials have been used regularly in all aspects of daily life. The lack of suitable destination for these materials can be a worldwide environmental problem, especially because plastic materials can take hundreds of years to decompose [1]. On the other hand, the reuse of waste by efficient recycling provides social, environmental, and economic gains and at the local level, it ensures money savings for communities [2]. Also, plastic recycling and demolition waste annually lead to abundant environmental and economic benefits using them as an alternative material in sustainable construction. Besides that, it can promote the solution of sanitary landfill problems and the reduction of carbon emissions [3].

Originally, virgin plastic production is mainly manufactured in Asia (50%), China (29%), North America (18%) and Europe (19%) [4]. Likewise, for example, in the USA the most common plastic wastes that have been streamed include high-density polyethylene (HDPE), low-density polyethylene (LDPE), ethyl vinyl acetate (EVA) and polyvinyl chloride (PVC) [1]. 

In addition, polypropylene (PP) and high-density polyethylene (HDPE) are relevant polymer wastes because they are polyolefins used in foam sheets and can indicate satisfactory results even with 50% of granules replacing virgin materials [5].

Moreover, PP and HDPE can be reused without getting changes which are known as primary recycling [6]. Nonetheless, they should have been cleaned and uncontaminated [7]. Mechanical recycling can also be used by extrusion, injection, thermoforming, compression molding and rotational molding. The polymeric material is usually in powder form after the revaluation process (collection, cleaning, drying and grinding). In this method, the degradation of the material occurs after each reprocessing cycle and its advantage is the recycling of contaminated material after the washing process [8]. Therefore, contaminated plastic waste and different types of mixed material result in a higher cost in recycling than the incineration method. One of the most used materials in mechanical recycling is polyethylene (HDPE, LDPE, LLDPE) [9]. This thermoplastic polymer is used in rotational molding due to their mechanical properties and costs [10].

In the chemical recycling or tertiary recycling, the polymer chains are transformed into smaller molecules through chemical processes such as hydrolysis and pyrolysis. In this process, the plastic waste can be processed back to produce basic petrochemicals for making virgin plastic or refined fuels [11,12]. In spite of this, plastic waste consumes high energy costs. Either recycling of plastic waste by primary or secondary methods causes degradation of properties. Thus, the reprocessing of materials can lead to producing inferior and low-quality products.

Similarly, chemical recycling, depending on the material, is not efficient enough or economically sustainable. Therefore, in many cases, the mechanical properties are used in some applications such as filling or incineration and roads [13,14]. It should be noted that studies were carried out on the addition of rubber to bricks, obtaining results similar to conventional ones, providing the use of this material in small proportions without significant damage to its properties. It may be considered feasible to add this material as a sustainable alternative, reducing natural resources and inserting discarded resources into the environment [15]. The other method is upcycling, which makes available the reuse of waste materials in an increased product, adding again the consumption cycle [14].

Moreover, in the recycling process, there are some obstacles, as energy consumption, costs in the separation of materials and differences in useful life between virgin and recycled materials. Therefore, it is necessary to review the benefit rates of the methods used for recycling [16]. At the same time, in collaborating with the environment, the use of recycled reticulated HDPE powder obtained from rotomolded parts by shearing in the solid-state, presented a melting behavior similar to that of virgin material, using the compression molding process [17].

It is estimated that around 98,000 t of polyethylene was on the market in 2018, and only 2% of this amount was destined for the rot molding process [10]. A worrying number is due to the dependence on polyethylene, with a percentage of 95% of the material used in rotational molding and 5% of the other materials [18]. The designs that use the rotational molding process to create their parts are dependent on polyethylene because it is more resistant to the ultraviolet (UV) rays, less porous and easy to clean. This allows low cost with eco-friendly products and more recyclable [19].

In 2018 ten events on rotational molding took place on five continents with a thousand participants. In this way, the world community of molders is thriving through specific communication networks, strengthening the community by sharing information and latest technologies to six continents. The number of molders has growing in recent decades and changes have taken place in regions in countries such as India, China, and Brazil. An estimated number of companies specializing in rotational molding is established in South America, North America, Europe, Africa and Asia [10].

Thermo-oxidative aging of polyethylene in the rotational molding process, the use of antioxidants in polyethylene has been proposed, resulting in a wide temperature range before the antioxidants are consumed in the rotational molding process [20]. The use of polyethylene with natural fiber through the rotational molding process shows some difficulties at the fusion of materials. Then, plasma treatment was proposed on the surface of the polymer, causing an upgrade in the union of materials, mechanical properties and humidity resistance [21]. Through Solidworks computational tools and computer-aided design (CAD) it is possible to model and simulate a rotational molding machine to provide long-term tasks, measurements of mold positioning between the axe and real distribution of the material in the mold [22]. In the production of a composite based on polycaprolactone (PCL) and reinforced with hollow glass microspheres, it is possible to see this application by rotational molding process with mechanical reinforcement, weight reduction and aesthetic improvement [23].

There are some variables in the rotational molding, as temperature, heating time and cooling model. These significant factors cause impacts on part quality [24].

With the concern to reducing environmental impacts and improving the quality of life, walls were created based on polyethylene terephthalate (PET) bottles filled with disposable materials such as paper, plastic bags, sand, and earth [25]. The ByFusion company called ByBlock changed waste in alternative blocks civil construction, without the need for adhesives or glues. This possibility reduced the emission of greenhouse gases by 95% compared to concrete [26].

Researches of plastic blocks developed in the United Kingdom called Everblock, obtained clear walls and made possible for the participant not to be trapped between the walls when applying the test [27].

The company Far Eastern Group developed the EcoARK building, with its facade composed of hollow blocks of recycled PET called Polli-Bricks. The material was reprocessed with a design that allows the fit between them, mounted in rectangular panels and coated with a film resistant to fire and water, being resistant to earthquakes, strong winds, and typhoons [28].

Another type of plastic block was named polyvinyl chloride concrete (PVC), guaranteeing the advantages of the polymeric material, being recyclable, resistant to rain, sea wind, immune to the action of fungi, bacteria, insects, most chemical reagents. This material has 20% of reduction of cost compared to the conventional masonry [29].

The company, named Conceptos Plásticos designed the BloquePas block, with the intention of replacing informal and precarious housing settlements with decent housing, helping to minimize environmental impacts caused by plastic waste. It offered 30% of lower cost incentives than the traditional buildings in rural locations [30].

The use of thermoplastic residues added to bricks encourages sustainable recycling, providing light, porous bricks with high thermal resistance, characteristics favorable to civil construction. Depending on the percentage of plastic material, its thermal resistance improves due to the number of void spaces [31]. HDPE polymer does not only have advantages. In the presence of high temperatures, the deformation of the material may occur, presenting high flammability presenting smoke. Thus, it is essential to insert material with a flame retardant effect in order to comply with government regulations that allow it to be introduced on the market [32]. PP has no toxic properties and is simple of processing [33]. However, PP consists mainly of carbon and hydrogen elements and is extremely flammable. In its burning process, PP causes the formation of dripping flames and smoke [34]. Thus, there is a need for the addition of flame retardant to meet certain applications [35]. 

The aim of this paper is the development, manufacture, and characterization of PP and HDPE recycled polyolefin blocks as masonry components in civil construction. These blocks were manufactured by the rotational molding process. Besides this, the mechanical, physical, impact and flammability properties of the blocks were studied.

Furthermore, the innovation of the study was the creation of an ecological block of 100% recyclable plastic and with high elastic recovery of HDPE blocks after compression tests. These materials can generate income, have a blue footprint, as they do not require water in their installation, provide agility in the installation, increase the life cycle of polymers, do not proliferate fungi and the standardization of the process of construction.

Likewise, in view of the concept of sustainability, this type of production only brings benefits to the environment, because it is estimated that in Brazil about 49% of plastic packaging is discarded and definitely becomes waste. Then, the result demonstrated in this work, removes from nature, when produced on a large scale, a large amount of waste, contributing to sustainability in the construction industry.

## 2. Materials and Methods

### 2.1. Materials Used

**Figure 1:**
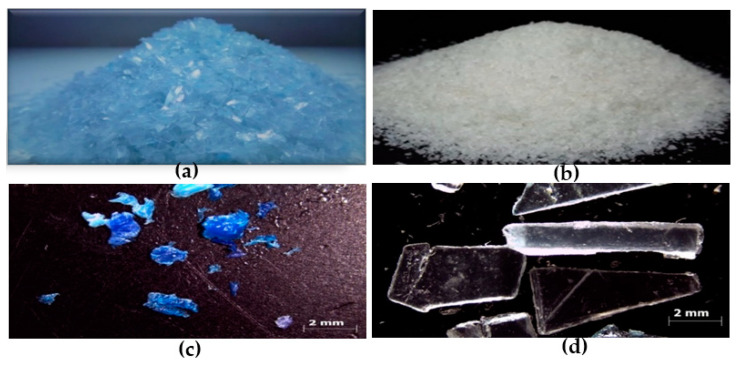
Polymers used: (**a**,**c**) polypropylene (PP) and (**b**,**d**) high-density polyethylene (HDPE) after cut by knife.

Post-consumer packaging of Polypropylene (PP) and high-density polyethylene were obtained from mineral water bottles and alcohol bottles-HDPE, respectively. These materials were obtained from laboratory and recycling cooperative of University Federal of Pará-UFPA, Belém, state of Pará, Brazil. These bottles were cleaned with water and neutral soap to eliminate impurities and remove labels. After drying at the temperature room, the bottles were subjected to cutting by knives (Model NFA-1533, manufacturer RONE and Cróton TE-625, manufacturer TECNAL) from UFPA, Belém, state of Pará, Brazil. This resulted in particles of different sizes and shapes (Figure 1).

The calcined alumina used was originated by ALUNORTE—Alumina do Norte S.A. company, from Belém, state of Pará, Brazil. The alumina is less stable than alumina-α. This type of alumina is used as raw material for the aluminum production process, as it uses less energy than its more stable form, called alumina-α.

### 2.2. Three-Dimensional Modeling of Molds for Manufacture of Polymeric Blocks

Firstly, the three-dimensional modeling of the blocks and molds was performed using computational resources from the SketchUp PRO 2017 software, version 17.0.18899 64-bit for the construction of the desired geometries and 3D visualization [36,37].

After this, the stainless-steel mold was made using a sheet with thickness of 3 mm, 2 thread bars of 9.53 mm in diameter with 350 mm and 400 mm of length, high temperature aluminum tapes to aid in sealing, nuts and alleys to fix the mold on the bars. At the aluminum mold, a plate with a single thickness of 1.5 mm, thread bars, nuts and washers were used.

Besides that, a rotational molding machine prototype was used to manufacturing the civil construction blocks. The mold was fed with 1.530 kg of material and the speeds of biaxial rotation were 6 rpm and 9 rpm (translation). The heating time was 50 min and cooling time was 30 min with biaxial rotation up to 60 °C for demolding the block [38].

### 2.3. Methods for Characterization HDPE and PP Materials

The optical microscope, Zeiss Axiocam 105 model, from UFPA, Belém, state of Pará, Brazil, was used for obtaining images of PP and HDPE post-consumer polymers. The Image J software version 1.52b was used to calculate the average particle size of polymers [39].

The density measurements (D) were carried followed by ASTM D792 standard [40,41] at the temperature of 23 °C. The density of the samples was determined by Equation (1):

D = a/(a + w − b) (1)

where:

a = apparent mass of specimen, without wire or sinker, in air,

b = apparent mass of specimen (and of sinker, if used) completely immersed and of the wire partially immersed in liquid, and

w = apparent mass of totally immersed sinker (if used) and of partially immersed wire.

X-ray diffraction (XRD) analysis was performed with a Bruker D2 Phaser diffracto-meter at room temperature, from UFPA, Belém, state of Pará, Brazil. The parameters used were: Cu radiation (Kα = 1.540598 Å), 30 kV voltage, 10 mA current, angular scanning range (°2θ) = 5–60° with Ni Kβ filter, at 0.02° angular step and 0.3 s per step time [42,43,44,45].

#### Blocks Characterization Tests

The three-point bending strength tests, following the ASTM D790-17 standard, at the strain rate of 10 mm·mim^−1^ [46], 5 mm·mim^−1^ [47] and 2 mm·mim^−1^ [48] were realized. The rotomolded specimens had 12.7 mm wide, 3.2 mm thick and 127 mm long [49]. Tests were performed on the EMIC model DL-500 universal testing machine with 12.7 mm diameter supports, from UFPA, Belém, state of Pará, Brazil. Seven specimens for each study condition were tested.

The impact strength test was performed by Gunt Hamburg model WP 410, from UFPA, Belém, state of Pará, Brazil. This equipment has a capacity of 300 Nm, hammer weight of 19.8 kg, impact velocity of 5.5 m·s^−1^, length of the pendulum of 840 mm, drop angle of 150° and supports for samples with an opening of 40 mm. It used the measures of the specimens and the procedures for execution of the Charpy impact test [21,50,51]. There was no used notch in the specimen.

In Brazil, there are no standards for polymeric modular building blocks. So, the polymeric blocks were characterized similarly to ceramic blocks. Thus, geometric characteristics (dimensions of the faces, thickness of the septa, external walls, deviation from the square and flatness of the faces) [52], mechanical characteristics (compression strength) [53], water absorption test were realized by ASTM C20-00 and ABNT NBR 15270-1 and ABNT NBR 15270-2 Brazilian standards [52,53,54,55]. Water absorption was a relation between the dry and wet specimen weights and Equation (2) was used:

Aw = (Ms − Md)/Md × 100 (%)(2)

where:

Aw is water absorption percentual;

Ms is saturated with water specimen masses;

Md is dry specimen mass.

The flammability properties were tested under vertical UL94 standard burning conditions for observations of the dripping phenomenon. In the experiment, the flame was applied to each specimen for 10 s and removed. Furthermore, once the combustion was extinguished the flame was applied again for 10 s [32,56,57]. The samples were cut from the fabricated PP and HDPE blocks with 5% of alumina filler and without fillers.

In Brazil, the Brazilian standard ABN NBT 15575-1 (2021) establishes fire safety of building components and building systems, in particular sealing systems and walls systems [58]. Moreover, plastics are flammable materials when heated and physical-chemical changes occur by which the materials decompose into volatile products. So, the main focus of the flammability was to evaluate if a certain polymeric material will have the required behavior. The flammability test follows the IEC 60695-11-10, ASTM D635 and UL94 international standards that evaluate points, such as: time to combustion, how long it took to extinguish combustion without intervention, if combustion continues and other tests that will determine the flammability potential and its resistance to flame exposure.

## 3. Results and Discussion

### 3.1. Three-Dimensional Modeling

**Figure 2:**
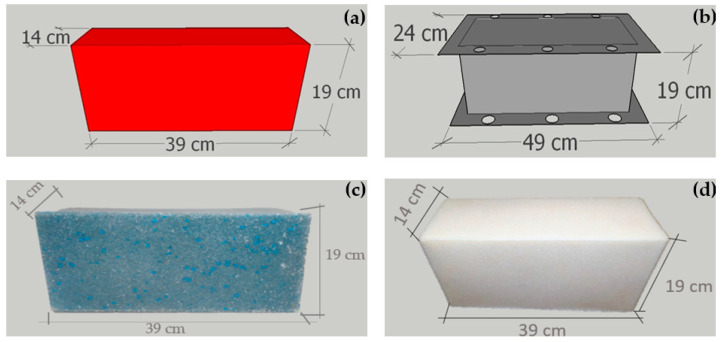
Modeling, molds, and blocks: (**a**) modeling the rotomolded block; (**b**) modeling the mold; (**c**) PP rotomolded block; (**d**) HDPE rotomolded block.

Figure 2 shows the modeling of the rotomolded blocks and the modeling of the molds. Initially, the stainless-steel mold was thought of as an option for the material to the molds. However, due to local limitations of material and laboratory, the molds were made with aluminum and blocks without interlocking. So, the stainless-steel mold was able to achieve its purpose which was to develop the rotomolded parts.

### 3.2. Polymeric Blocks Produced

The designed bars were effective in fixing the mold on the spider and the mold sealing with the help of high-temperature aluminum tape prevented material runoff. Despite the mold having an angle inclination in the male and female cavities, there was a small difficulty in the demold of the female cavity. The PP blocks had a surface finish with a discrete gloss and voids in their surfaces in the male cavities and in the upper corners acquired from the stainless-steel mold. The aluminum mold contributed to the surface finishing with accentuated shine, reduced number of voids on its surfaces, and similar porosity in both materials, however, it presented a regular surface with final surface quality.

The aluminum mold had no material run off and a 9 kg reduction in mold mass occurred. There was no difficulty in demolding, with the need to disassemble only the upper or lower part, making the part slide over the mold, facilitating the demolding of the rotomolded block.

The blocks had dimensions of 39 cm in length, 19 cm in height and 14 cm in thickness (width) (Figure 2).

The HDPE blocks show a surface with moderate gloss and its porosity was reduced by stainless-steel molds. The aluminum mold contributed to a smooth surface with high gloss and reduced porosity compared to the other developed blocks, there were no material dispositions, closing all corners in both metals used in Figure 2c PP rotomolded block and Figure 2d HDPE rotomolded block. As a smaller particle size material was used, a better sintering propagation was obtained, producing flat and regular surfaces with final surface quality.

The blocks made by PP had more production more economical than HDPE blocks, because they do not require a second grinding in the regrind of the polymer, reducing the final cost. They can be used with or without finishing materials such as glass tablets and metal profiles, because of their low porosity and smooth surface and overlapping regardless of the material used for the finishing.

#### 3.2.1. Optical Microscopy

**Figure 3:**
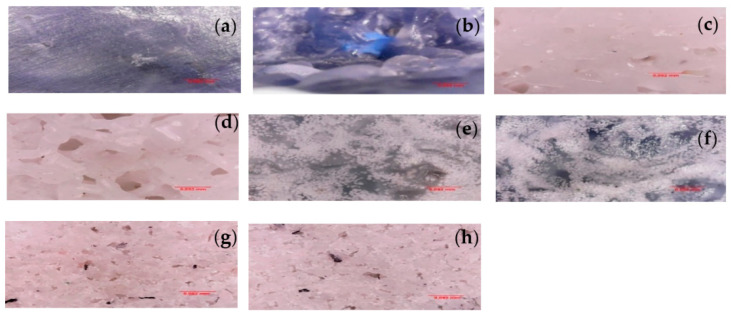
Transmitting light microscopy images of the blocks: (**a**,**b**) external and internal surface of the PP block, (**c**,**d**) external and internal surface of the HDPE block, (**e**,**f**) external and internal surface of the PP block with alumina and (**g**,**h**) external and internal surface of the HDPE block with alumina.

From Optical microscopy, no bubbles were identified in the blocks, which are the main defects reported for the rotomolding process. This fact indicates that the technique used in the processing was efficient [21]. Both materials show comparable thermal behavior. The polypropylene block showed porosity and texturing on the outer surface and great roughness on the inner surface in Figure 3a,b,e,f. The polyethylene pieces showed little porosity and a smooth outer surface and less roughness and a tangle on the inner surface in Figure 3c,d,g,h.

In the case of semicrystalline polymers, the cooling rate causes more effect on the crystallization phenomenon and the growth rate of the spherulites and their size [24]. The particles have different shapes after revaluation of the PP particles. So, the average area value of PP was calculated and equal value of 10.15 mm and for the HDPE particles, the average area value of HDPE was 1.53 mm. A wide particle size distribution compensates for the low packing density of large particles and leads to smaller bubble sizes in the melt [59]. However, the irregular particle shape decreases the sintering efficiency in PP, leading to a lesser efficient sintering process than HDPE [60].

#### 3.2.2. Density

The results of the density analysis by the pycnometer method, performed at the temperature of 27 °C of distilled water with its density of 1.0 g/m^3^, show that the revalued post-consumer packaging presented values of 1.0 g.m^−3^ for PP and 1.0 g.m^−3^ for HDPE. Therefore, with the results obtained, it was possible to measure and to compare the values established by ASTM D792 standard. Thus, the values obtained were analogous in with the standard. This standard informs values independent of virgin or processed materials, showing that there was no adulteration with cheaper material and ensuring the homogeneity of the rotomolded materials [41]. As the PP and HDPE polymers used have similar densities, the same amount of material was used in the molds, standardizing the block development process.

#### 3.2.3. X-ray Diffraction of Post-Consumer and Rotational Molded PP e HDPE

**Figure 4:**
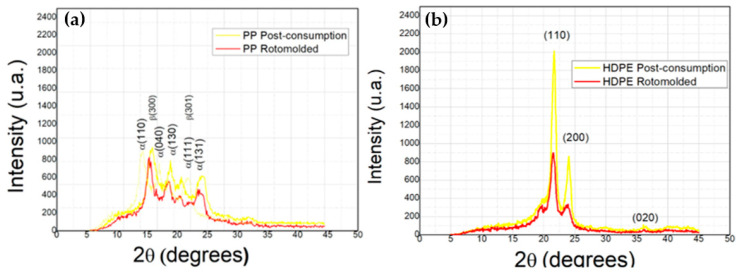
Diffractograms: (**a**) post-consumer and rotomolded PP material; (**b**) post-consumer and rotomolded HDPE material.

**Figure 5:**
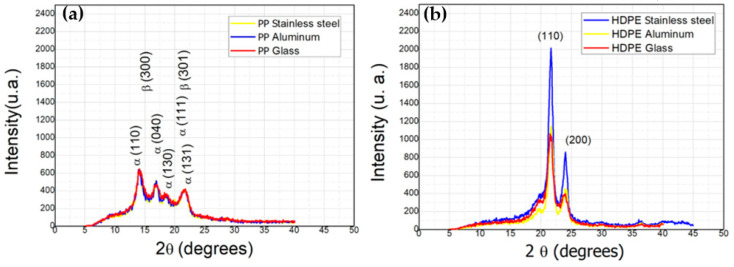
Diffractograms: (**a**) PP rotomolded material processed with stainless steel, aluminum, and glass mold; (**b**) HDPE rotomolded material processed with stainless steel, aluminum, and glass mold.

Figure 4 shows the PP post-consumer diffractogram and rot-molded PP material. Figure 5 presents PP rotationally molded with stainless steel, aluminum and glass molds with the characteristic peaks of the α phase PP crystal centered at angles 2θ = 14.0°, 17.0°, 18.5°, 21.0° and 22.0° [61,62,63,64]. Corresponding to the α (110), α (040), α (130), α (111) and α (131) planes and PP crystalline β phase, it finds reflections centered at the 16° and 21° angles corresponding to the β(300) and β(301) associated with the crystal configuration equivalent to PP [65,66,67]. 

The HDPE samples obtained in Figure 4b from the diffractogram of post-consumer and rot-molded material and Figure 5b roto-molded HDPE with stainless steel, aluminum and glass molds, it can observe two refraction peaks at the 22.00°, 24.27° and 36.20°. These peaks are associated with the HDPE equivalent crystal structure related to the crystallographic planes (110), (200) and (20) [68,69,70]. In both samples of rotomolded PP and HDPE polymers, there was a difference in the diffraction intensity of the peaks compared to the post-consumer ones, resulting in a reduction in intensity and to indicating that this disparity caused changes in crystallinity [71]. Therefore, a shift to smaller angles occurs, indicating the effect of thermomechanical degradation on crystal morphology [72]. So, solving technical problems with the rotational molding machine, three types of molds were developed to obtain a mass reduction in the mold. 

Moreover, it can analyze rotomolded parts with aluminum, stainless steel, and glass mold. Thus, it was observed that there was no expressive change in the peak intensities in the PP samples in Figure 5a. Besides that, the same did not occur with HDPE samples in which the stainless-steel mold showed a disparity compared to the other analyzed molds Figure 5b. The mold cooling time may have influenced the HDPE recrystallization.

### 3.3. Mechanical Behavior and Characterization of Masonry Blocks

#### 3.3.1. Flexural Strength

It was calculated the modulus of elasticity (E) and maximum strain (ε) from the flexural test. It was shown 302 MPa for PP with 2 mm·min^−1^ (PP2), 266 MPa for 5 mm·min^−1^ (PP5) and 83 MPa for 10 mm·min^−1^ (PP10) for the respective 22 mm, 19 mm and 20 mm. I addition to, the values of 91 MPa for HDPE in 2 mm·min^−1^ (HDPE2), 76 MPa for 5 mm·min^−1^ (HDPE5) and 26 MPa for 10 mm·min^−1^ were obtained (PEAD10) for the modulus of elasticity in respective 26 mm, 22 mm and 19 mm for strain ((ε) reported in Table 1.

**Figure 6:**
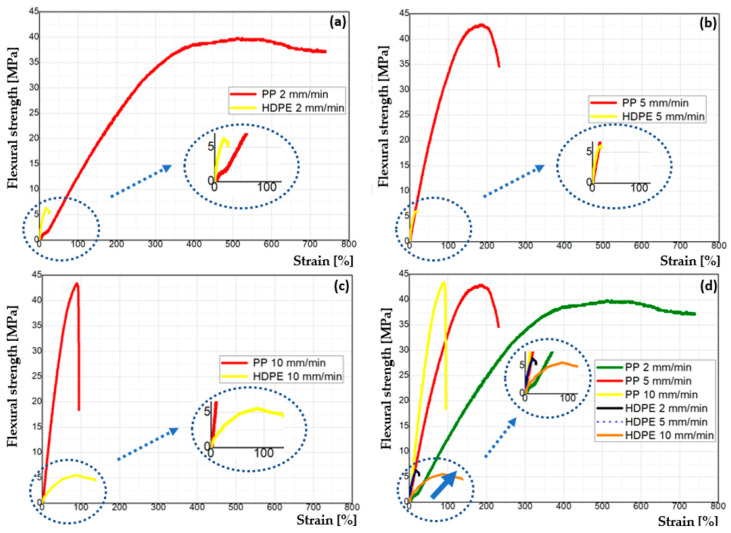
Flexural strength versus deformation (strain) curves: (**a**) PP and HDPE at the strain rate of 2 mm·min^−1^; (**b**) PP and HDPE at the strain rate of 5 mm·min^−1^ (**c**) PP and HDPE at the strain rate of 10 mm·min^−1^ and (**d**) PP and HDPE at the several strain rate (from 2 mm·min^−1^ to 10 mm·min^−1^).

It is observed that the sample PP exhibits modulus of elasticity values higher than HDPE at the several strain rates studied. As expected, the strain rates change the flexural strengths and modulus of elasticities, in especial for the PP material. With the increase of rates, the E was reduced. Thus, a variation was observed under the influence of the strain rate applied in the tests illustrated in Figure 6.

It was observed that PP2 samples showed highest flexural strength of blocks tested. 

In the flexural test, the samples of PP2 from literature showed better results with materials recycled by rotational molding, now according to a work performed by rotational molding with virgin polyethylene and was performed the flexion test of the external surface, showed a value close to 17.5 MPa [73]. Despite being the same process, the samples of the rotationally molded blocks have porosity and interstitial spaces between the structures, facilitating the agglutination of other materials on the external surfaces of the blocks, such as cement and external and internal finishing materials in walls.

#### 3.3.2. Impact Test

**Figure 7:**
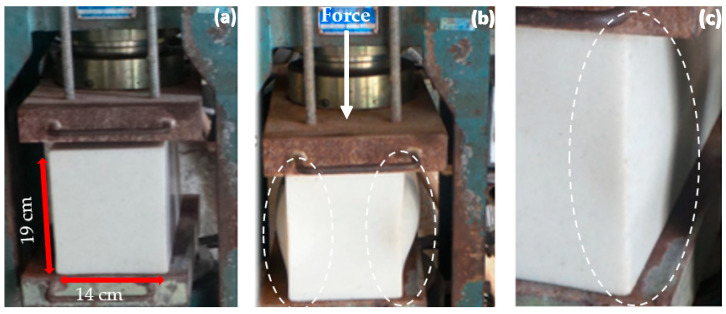
Compression strength test: (**a**) the block at the beginning of the test; (**b**) the block receiving a load and suffering a local elastic deformation on the sides of the block and (**c**) the block returning to its initial shape after the removal of the load applied on it.

The PP block showed a 0.62 kJ·m^−2^ value and the HDPE block presented a 2.48 kJ·m^−2^ value reported in Figure 7.

For HDPE pure the impact strength is 6 kJ·m^−2^ [21] and 3 kJ·m^−2^ for PP pure [50]. So, both types of polymers studied had a decrease of values of impact strength related to HDPE pure and PP pure. This behavior is expected because of the porosity of recycled blocks and reduction of intensity in the crystallographic planes of both recycled polymers. The reprocessing impacts the mechanical strengths [72].

#### 3.3.3. Geometric Characteristics

Determination of the face dimensions, effective dimensions, deviation from the square (D) and from the flatness of the faces (F) and thickness of the external walls and septa of the blocks.

In the test for determining the thickness of the external walls, it can observe the measurements of the measures of the block of PP with 40 mm and HDPE 44 mm corresponding to the values of the external septa met the minimum requirements, according to the tolerance of the sum of 20 mm minimum of the external septa in Table 2. Related to the determination of the measures of the faces, the measurements of width 136 mm for PP and 137 mm for HDPE, height 191 mm for PP and HDPE and length of 385 mm in both samples of PP and HDPE were obtained. The average tolerance was od ±3 mm for width, height and length.

In the test for determining deviations from the square, it can observe the measurements of the PP block with 1.5 mm and HDPE with 1 mm, to meeting the tolerance of 3 mm maximum. 

In the determination of the flatness of the faces, the values were for the blocks of PP 1.31 mm and HDPE 2 mm the average of the analyzed blocks met the minimum requirements of maximum tolerance of 3 mm deviation. Thus, the rotomolded blocks were accepted as to the analysis of the geometric characteristics not presenting deformities outside the requirements. Unlikely the certified and non-certified ceramic blocks for masonry sealing, commercialized in the region of Chapecó in the state of Santa Catarina, Brazil about 30% did not meet the requirements as to the geometric characteristics [52,53,74].

#### 3.3.4. Water Absorption Test

As expected there was no water absorption in PP and HDPE blocks. The Brazilian standards establish maximum values of 20–22% for ceramic blocks [52,53]. This property is interesting to avoid problems of lack of water tightness and presence of humidity in components or parts of edification.

As an example, PP and HDPE blocks with geopolymer showed water absorption from 13% to 20% [55]. The authors believe this difference is due to the size of particles of polymers.

#### 3.3.5. Compressive Strength

The PP block’s compressive strength is higher than the HDPE block’s compression strength, as can be seen in Table 2. So, the PP blocks showed a compression strength 50% higher than the HDPE blocks. It was found that the PP blocks after the test, suffered ruptures on the left and right sides with the ruptures similar to ceramic materials after the compression strength test observed in the study [75,76].

The HDPE blocks showed local elastic deformation, forming an appreciable lateral deformation not occurring rupture of the block, after the removal of the applied load. The samples tend to return to their initial shape, indicating that the HDPE blocks have a larger elastic phase than the PP ones. 

It can be observed in Figure 7a the block at the beginning of the test, in Figure 7b the block receiving a load and suffering a local elastic deformation on the sides of the block. In the Figure 7c, the block returned to its initial shape after the removal of the load applied on it and its recovery after the tests. This did cause a permanent residual deformation as in the study of [77] and indicated a longer elastic deformation than the PP block.

HDPE presents a ductile behavior when subjected to compression and its compressive strength is adjacent, when compared to the strength of concrete and wood. Therefore, the application of HDPE would be a robust structural element to be submitted to compression [78].

A steel mesh with a diameter of 3.4 mm was added to the blocks as reinforcement to increase the compressive strength. The Figure 7a shows a structure developed for the reinforcement of the blocks. Also, the Figure 7b exhibit the mold with the frame inside waiting for the material to be poured to explore a possible increase in compression strength.

**Figure 8:**
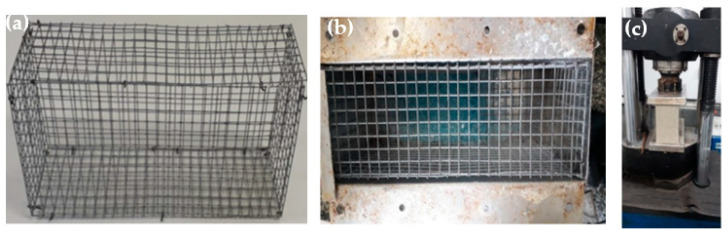
Block with reinforcing mesh: (**a**) block mesh; (**b**) mesh inside the mold and (**c**) block with the mesh in the test.

The HDPE blocks reinforced with the metal mesh showed better compressive strength than the HDPE blocks without reinforcement Figure 8c, indicating an increase of 0.3 MPa, with a maximum strength of 0.9 MPa and the PP blocks showed a reduction of 0.3 MPa exhibiting maximum strength of 0.7 MPa.

The filled PET bottle-based blocks can be a good alternative to solve the increase of compressive strength, by adding concrete inside the rotomolded blocks. Another solution would be to add sisal or cabuya fiber changing the elastic properties of the matrix polymer resulting in lower plastic deformation and higher elasticity, accompanied by shorter recovery times [77]. 

Although the rotomolded blocks do not meet the requirements corresponding to the mechanical strength according to the standard adapted for ceramic blocks.

#### 3.3.6. UL 94 Vertical Flammability Test

**Figure 9:**
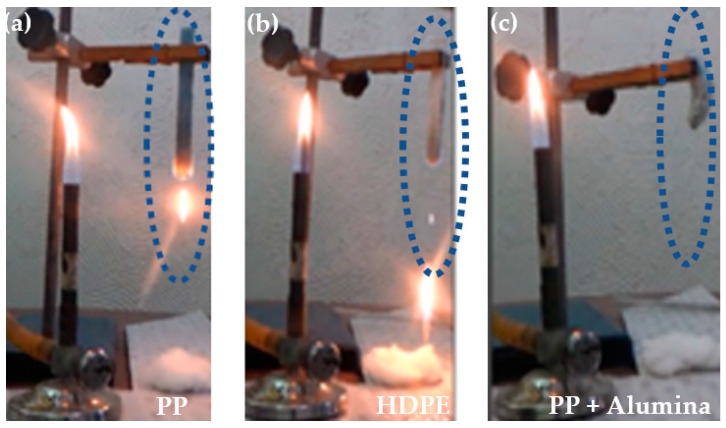
Flammability test after 10 seconds removal from the flame: (**a**) PP; (**b**) HDPE; (**c**) PP + 5% alumina.

It was observed that the samples without the incorporation of alumina in the blocks, suffer ignition followed by a flaming drip on the cotton in the second application of the flame to the specimen, characterizing it as the materials with the classification according to UL 94 Vertical of V-2 (Figure 9a,b). 

**Figure 10:**
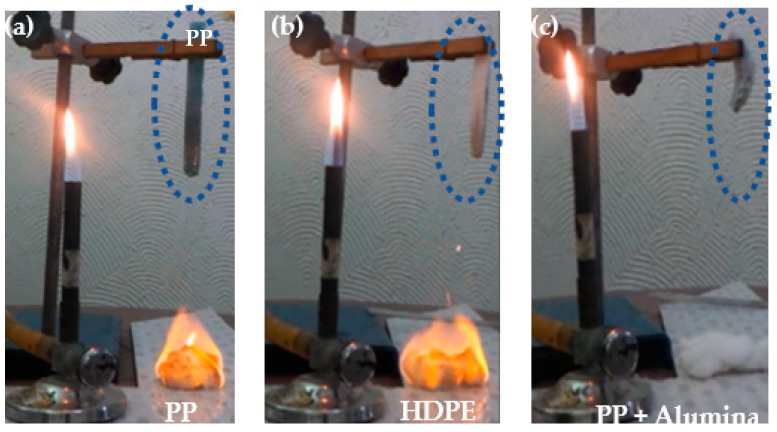
Cotton fiber results after the flammability test: (**a**) PP; (**b**) HDPE; (**c**) PP + 5% alumina.

In Figure 10a the PP sample without flames and the cotton fiber with flames after contexture and in Figure 10b the same occurred with HDPE, characterizing the two materials PP and HDPE as V-2.

However, in the samples with 5% incorporation of alumina, the flame retardant in the HDPE and PP blocks in the first application of the flame for 10 s, the set of specimens sustains the flame for only 5 s and goes out. After this, in the second application of the flame for 10 s the flame sustains for only 5 s and goes out, with no dripping of the material on the cotton. Thus, reaching the UL-94 vertical classification as V-0, referring to the classification requirements and in Figure 10c the PP sample with 5% alumina without dripping. Figure 9c illustrates the cotton fiber without flame due to the material being classified as V-0. 

### 3.4. Technical, Economic, Environmental Feasibility, and Industrial Scale Production

One of the main advantages of reuse in civil construction is the reduction of non-renewable raw materials, such as aggregates and materials in the manufacture of cement. This optimizes the environmental damage caused by the extraction of raw materials and also represents a lower financial cost, since the material of these recycled polyolefins would be a raw material that is discarded every day as waste. That is why there is a growing interest in studying and improving the research on the incorporation of such material, thus providing security for the use of blocks and developing the interest of other companies to increase and invest in the recycling sector. 

Therefore, when we observe the results of the large-scale manufacturing of recycled polyolefin blocks compared to conventional blocks, we conclude that there is a considerable financial advantage, especially if the company obtains the residue as a donation. 

The results obtained with the characterization tests are difficult to obtain similarities if there is no similarity of the methods used in this present paper. Due to the difficulty of numerous tests with rotomolded parts with several variables to be analyzed such as rotation speed, material size and translation, heating time and amount of material.

The notable results in this study were the achievement of filling the corners of the pieces, as they are not rounded, which makes it difficult to fill in the rotational molding process and total elastic recovery of the HDPE pieces, after the compression tests. 

Also, using the standardization that was used in this study, it will be possible to perform repeatability, we recommend for future studies the use of an industrial scale rotational molding machine, since the one used in this study was a laboratory scale, which brought some limitations and allowed the production of a part in each cycle. If an adaptation is made that makes it possible to store several molds in the machine’s oven, it would facilitate the production of the parts and, as a consequence, the tests. We indicate the use of a thicker aluminum plate for making the molds, as the one used in this study needed some care in the release process so as not to cause damage to the mold due to the thickness of the plate according to the release of several pieces.

## 4. Conclusions

Conclusions can be drawn:

- It was possible to produce the blocks with PP and HDPE materials, increasing the life cycle of the packaging by rotational molding.
- Aluminum mold proved to major material and it reached a reduction of 9 kg of the total mold weight and offered no difficulties in the demold.
- The compressive strength of HDPE rotomolded blocks with 1.53 mm particle diameter was 0.6 MPa and PP blocks with 10.15 mm particle diameter were 0.9 MPa. Therefore, HDPE block would be an excellent material for use in modular construction, in special considering the lightweight material in comparison with traditional ones (ceramic).
- In order for the rotomolded blocks to be applied in civil construction they need to pass a flammability test, so that in case of fire, the fire does not spread through the dripping of the pieces. Therefore, the blocks with the addition of 5% alumina as fire-retardant obtained a V-0 classification. Thus, obeying the requirements of the vertical flammability test of the ASTM UL 94 standard can be used in civil construction.
- The increase in the speed of the flexural tests has a great influence on the results obtained in this study. The results obtained show that there was a reduction in the extracted values with increasing speed in the flexural tests.

## Figures and Tables

**Table 1:** Flexural Results.

Properties	Strain Rates Used		
	2 mm·min^−1^	5 mm·min^−1^	10 mm·min^−1^
E (modulus of elasticity)-PP	302 MPa	266 MPa	83 MPa
ε ( maximum strain)-PP	22 mm	19 mm	20 mm
σ (flexural strength)-PP	34 MPa	27 MPa	9 MPa
E (modulus of elasticity)-HDPE	91 MPa	76 MPa	26 MPa
ε (maximum strain)-HDPE	26 mm	22 mm	19 mm
σ (flexural strength)-HDPE	8 MPa	8 MPa	2 MPa

**Table 2:** Compressive Results.

Number of Blocks (13)	Septum Sums		Width (mm)		Height (mm)		Length (mm)		D (mm) ^(1)^		F (mm) ^(2)^		Compression (MPa)	
	PP	HDPE	PP	HDPE	PP	HDPE	PP	HDPE	PP	HDPE	PP	HDPE	PP	HDPE
µ (mean)	40	44	136	137	191	191	385	385	2	1	1	2	1	1
Sd ^(3)^	0	0	0.5	0.0	0.5	0.0	0.0	0.0	0.0	0.0	0.5	0.0	0.0	0.0

^(1)^: Deviation from the square (D); ^(2)^: Flatness of the faces (F); ^(3)^: Sd: Standard deviation.
